# The role of nephrologists in management of hypokalemic periodic paralysis: a case report

**DOI:** 10.1186/s13256-022-03283-0

**Published:** 2022-02-11

**Authors:** Julia Li, Suha Moten, Anis A. Rauf

**Affiliations:** 1 Midwestern University, Chicago College of Osteopathic Medicine, Downers Grove, IL 60515 USA; 2 Midwestern University, MABS, Downers Grove, IL USA; 3 Nephrology Associates of Northern Illinois and Indiana, Hinsdale, IL USA

**Keywords:** Case reports, Hypokalemic periodic paralysis, Nephrology, Dichlorphenamide

## Abstract

**Background:**

Hypokalemic periodic paralysis is a chronic condition characterized by sporadic attacks of weakness associated with acute hypokalemia. Attacks are typically associated with specific triggers, such as prolonged rest following exercise or consumption of a high-carbohydrate meal. Most commonly, this condition is caused by an autosomal dominant calcium channel mutation, and patients typically have an established family medical history of hypokalemic periodic paralysis. Long-term complications include the development of progressive proximal myopathy. Oral potassium chloride may be considered for the treatment of an acute attack, with administration of acetazolamide or dichlorphenamide as long-term prophylaxis. Nephrologists can play an important role in the recognition and treatment of previously undiagnosed hypokalemic periodic paralysis.

**Case presentation:**

We summarize the case of a 19-year-old white man who presented to the emergency department with undiagnosed attacks of hypokalemic periodic paralysis, and who reported, at follow-up, improvement in the severity and frequency of attacks with dichlorphenamide.

**Conclusions:**

This case demonstrates the crucial role nephrologists can play, not only in the diagnosis of hypokalemic periodic paralysis, but also in the ongoing management of this condition. Patients should be advised to regularly follow up with their nephrology team for evaluation due to the risk of developing myopathy.

## Background

Periodic paralyses are a rare group of disorders characterized by sudden and sporadic episodes of flaccid paralysis [1]. Hypokalemic periodic paralysis, the most commonly occurring subtype, has an estimated prevalence of 1:100,000 [1]. Onset of hypokalemic periodic paralysis typically occurs between 5 and 35 years of age, with most attacks occurring between 15 and 35 years of age [1, 2]. The frequency of attacks typically decreases after 40 years of age [3]. Attacks involve episodes of muscle weakness, often localized proximally, in the lower limbs, which occur sporadically with a duration ranging from hours to several days [2, 4]. Attacks typically occur in the early morning or in the middle of the night [3]. Common triggers include rest after prolonged exercise and carbohydrate-rich meals [3]. Hypokalemic periodic paralysis is an autosomal dominant inherited genetic disorder [1]. Indeed, two of three missense mutations of the gene coding the alpha subunit of the skeletal muscle L-type calcium channel (*CACN1AS*) are associated with ~70% of cases of hypokalemic periodic paralysis; a point mutation in the skeletal muscle sodium channel (*SCN4A*) gene accounts for 10–20% of cases [3].

Patients with severe hypokalemia may experience respiratory failure due to paralysis, which can be fatal [5]. Most patients eventually develop progressive proximal myopathy, most severely in the proximal limbs and pelvic girdle [2, 6]. However, development of myopathy is not associated with the number or severity of weakness episodes during the lifetime of a patient [2]. Given the impact of hypokalemic periodic paralysis attacks on patients, proper diagnosis and management of this condition is paramount [4]. We present a case report to highlight the important role of nephrologists in the recognition and treatment of previously undiagnosed hypokalemic periodic paralysis.

## Case presentation

**Fig. 1:**
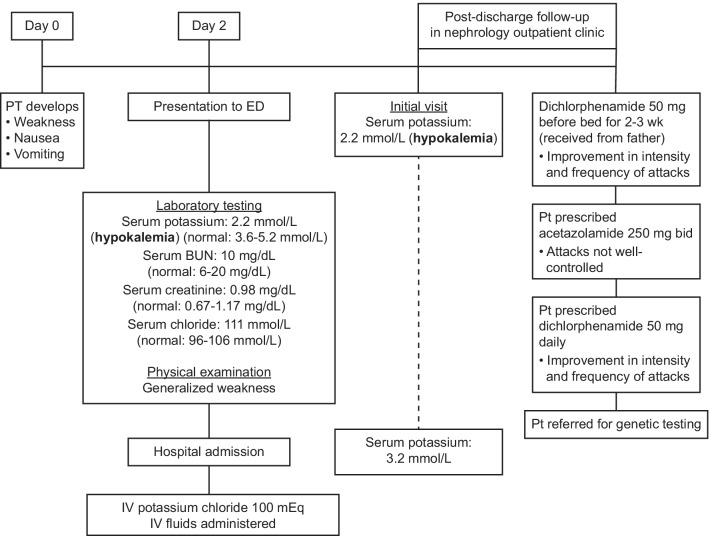
Timeline of patient visits. *bid* twice daily, *BUN* blood urea nitrogen, *ED* emergency department, *IV* intravenous, *pt* patient

A 19-year-old white man presented to the emergency department (ED) for evaluation of weakness, nausea, and vomiting that began 2 days prior (Fig. 1). The patient had a personal medical history of chronic hypokalemia and his father had been diagnosed with hypokalemic periodic paralysis; there was no other family history of renal or neurologic disease. Similar episodes had occurred since the onset of puberty at age 11 years. These episodes are presumed to be associated with exposure to cold weather, intense physical activity, or rest for prolonged periods, with the long-term history of hypokalemia. The patient reported occasional marijuana use and was previously counseled about the possibility of cannabinoid hyperemesis syndrome causing symptoms. He denied illicit drug, alcohol, or tobacco use. Laboratory testing was conducted in the ED, with the only markedly abnormal result being a serum potassium level of 2.2 mmol/L, indicating hypokalemia. This electrolyte imbalance resulted in a nephrology consult. Serum magnesium, chloride, and glucose levels were elevated (1.7 mg/dL, 111 mmol/L, and 104 mg/dL, respectively). Cannabinoid testing was positive. Serum blood urea nitrogen (10 mg/dL) and serum creatinine levels (0.98 mg/dL) were normal. Physical examination was notable only for generalized muscle weakness.

The patient was previously prescribed oral potassium chloride 20 mEq/15 mL daily during acute attacks, which improved weakness within ~1–2 hours. The patient reported previous hospitalizations for intravenous potassium chloride administration for attacks of weakness due to severe nausea, which prevented oral potassium chloride ingestion, as was the case during this current visit. The frequency of hypokalemic attacks and hospitalizations related to these attacks have increased with the patient’s age. The patient reported numerous hypokalemic attacks, with ten resulting in ED visits in the previous year. The patient presented with a potassium level of 1.8 mmol/L at one of these visits.

For the current attack, the patient’s condition improved with potassium repletion (100 mEq) and several liters of intravenous fluid over several days during hospitalization. His condition improved in 2 days, and he was advised to follow a high-potassium diet. During follow-up in the nephrology outpatient clinic, the patient presented with a low potassium level (Fig 1). Since hospital discharge, he began taking dichlorphenamide tablets, which he received from his father, 50 mg once daily at bedtime for 2–3 weeks. He noted less frequent and severe symptoms. Although he reported improvement with dichlorphenamide, his insurance required an initial trial of acetazolamide 250 mg twice daily, which he received at discharge. At this time, the patient noted that his father had previously received acetazolamide but experienced no symptom improvement, warranting a switch to dichlorphenamide. A few weeks later, the patient stated that his condition was not well controlled with acetazolamide, and he was prescribed dichlorphenamide 50 mg daily, which improved the intensity and frequency of weakness. In his most recent nephrology follow-up, the patient reported continued symptom improvement. At follow-up, his serum potassium level was 3.2 mmol/L. Genetic testing is pending.

## Discussion and conclusions

This case report underscores the important role nephrologists can play in the recognition and chronic management of hypokalemic periodic paralysis. Although this patient did not have abnormal kidney function or renal disease, nephrologists consulted for acute electrolyte management made the connection between the patient and family medical histories to establish the diagnosis of hypokalemic periodic paralysis. Given the association of hypokalemic periodic paralysis with potassium imbalance and acute attacks of weakness, this is a condition that nephrologists may encounter in clinical practice.

Differential diagnosis of hypokalemic periodic paralysis includes syndromes that cause acutely low potassium levels, including type I or type II renal tubular acidosis. Type I renal tubular acidosis is a distal tubular disorder marked by hydrogen ion secretion impairment, which can be caused by genetic defects, autoimmune conditions, or medications (e.g., lithium, amphotericin B) [7]. However, type I renal tubular acidosis can be distinguished from hypokalemic periodic paralysis by alkaline urine, despite a normal anion gap metabolic acidosis [7].

Type II renal tubular acidosis is a proximal tubular disorder characterized by impaired bicarbonate resorption [8]. Similar to type I renal tubular acidosis, patients experience normal anion gap metabolic acidosis; however, patients with this disorder can acidify urine [7, 8]. Patient medical history can be considered when distinguishing type II renal tubular acidosis from hypokalemic periodic paralysis. Type II renal tubular acidosis is associated with conditions that include cystinosis, Lowe’s syndrome, and Wilson’s disease, and with the use of some medications (i.e., cisplatin, topiramate, valproate) [9]. In contrast, hypokalemic periodic paralysis is associated with specific triggers and a strong family medical history [2].

Other differential diagnoses that need to be considered include other conditions characterized by muscle weakness (e.g., myasthenia gravis) [10]. However, unlike the episodic muscle weakness associated with hypokalemic periodic paralysis, the weakness associated with myasthenia gravis tends to be predictable in that it is typically preceded by exertion and, unlike hypokalemic periodic paralysis, involvement of extraocular and bulbar muscles is common [10, 11]. Other myopathies that can be considered include botulism, Guillain–Barré syndrome, and tick bite-related paralysis [10]. These myopathies can be differentiated from hypokalemic periodic paralysis by obtaining a thorough patient history (e.g., travel, known insect bite, sensory changes). It is also important to rule out hyperthyroidism, a potential cause of acquired hypokalemic periodic paralysis.[10]. In contrast to patients with familial hypokalemic periodic paralysis, patients with thyrotoxic periodic paralysis experience acute attacks during a hyperthyroid state, and symptoms discontinue once the hyperthyroidism is adequately treated.

Hypokalemic periodic paralysis should be considered in patients with a medical history of chronic hypokalemia or intermittent episodes of acute weakness associated with a trigger(s) [2]. Nephrologists should query patients regarding family medical history and previous hospitalizations for similar symptoms. Previous use of potassium chloride or patient familiarity with acetazolamide or dichlorphenamide may be indicative of hypokalemic periodic paralysis. Nephrologists who manage patients with hypokalemic periodic paralysis should recommend genetic counseling and ensure patients understand the heritability of the disease. Because most cases are attributed to an autosomal dominant mutation, patients of child-bearing age or those with children need to understand the increased likelihood of their offspring having the same condition. A review of the early-stage symptoms that may present is important to include in patient education and ongoing discourse with families. Additionally, nephrologists should emphasize the importance of routine follow-up visits for ongoing evaluation and disease management.

When patients present with muscle weakness, hypokalemia, and a family medical history of hypokalemic periodic paralysis, further diagnostic testing is not required. Genetic testing may not always be definitive due to potentially unidentified genetic mutations that cause hypokalemic periodic paralysis. Electromyography could demonstrate reduced compound muscle action potential during acute attacks or during an exercise test in which the electrical activity of the muscle is recorded [2, 10]. A decrease of ≥40% in compound muscle action potential amplitude from its maximum value during or after exercise is considered indicative of hypokalemic periodic paralysis [2].

Treatment of acute attacks of hypokalemic periodic paralysis includes oral potassium chloride 1 mEq/kg body weight per day, with the potential for an additional 0.2–0.4 mEq/kg body weight if patients do not experience improvement within 30 minutes (maximum, 200–250 mEq/day) [2]. For patients who cannot swallow oral tablets, intravenous potassium chloride may be administered [dosing not to exceed 40 mEq/L (maximum, 20 mEq/h and 200 mEq/d)] [2]. Because of the range of change in potassium levels that can occur during an acute attack of hypokalemic periodic paralysis, it is important to routinely monitor for electrocardiogram changes (e.g., ST-segment depression, U waves, and T-wave inversion and flattening) that are associated with hypokalemia.[12]

Acetazolamide, a carbonic anhydrase inhibitor that can be used as prophylactic therapy, is effective in approximately half of patients with hypokalemic periodic paralysis [2, 13]. It is hypothesized that the systemic acidosis created by facilitating sodium bicarbonate excretion can reduce susceptibility to attacks, although the mechanism is unclear [2]. Twice-daily dichlorphenamide, another carbonic anhydrase inhibitor, significantly decreased the median number of attacks, severity-weighted attack rate, and duration of attacks compared with placebo in a 9-week randomized, controlled study (*p* = 0.02, all comparisons) [14]. Dichlorphenamide significantly improved several aspects of patient quality of life compared with placebo [14]. Furthermore, efficacy was maintained with long-term (up to 1 year) open-label dichlorphenamide treatment [14]. Nephrolithiasis is a common adverse effect associated with acetazolamide and, rarely, with dichlorphenamide, and may warrant further management by nephrologists [2].

In conclusion, nephrologists play a key role in the diagnosis of hypokalemic periodic paralysis, a condition that is associated with multiple hospitalizations before patients receive appropriate treatment. Educating patients on the importance of genetic counseling and testing is critical. Furthermore, due to the risk of developing myopathy, it is critical that patients regularly follow up with their nephrology team for evaluation.

## Data Availability

Not applicable.

## Abbreviations

bid: Twice daily
BUN: Blood urea nitrogen
ED: Emergency department
IV: Intravenous
pt: Patient
